# A hemispheric comparison of cognitive dysfunction and sleep quality impairment in Middle Cerebral Artery infarction

**DOI:** 10.12669/pjms.36.3.1385

**Published:** 2020

**Authors:** Danyal Wahid, Hifza Rabbani, Ayesha Inam, Zubaa Akhtar

**Affiliations:** 1 Danyal Wahid, BS Hon. Department of Humanities, COMSATS University Islamabad, Islamabad Campus, Pakistan; 2 Hifza Rabbani, BS Hon. Department of Humanities, COMSATS University Islamabad, Islamabad Campus, Pakistan; 3 Dr. Ayesha Inam, PhD. Department of Humanities, COMSATS University Islamabad, Islamabad Campus, Pakistan; 4 Zubaa Akhtar, BS Hon. Department of Humanities, COMSATS University Islamabad, Islamabad Campus, Pakistan

**Keywords:** Cognitive dysfunction, Infarction, Middle cerebral artery, Sleep quality impairment

## Abstract

**Objectives:**

To investigate the severity of cognitive dysfunction and sleep quality impairment in patients with middle cerebral artery (MCA) strokes across the left and right hemisphere. Moreover, it also study gender differences with respect to MCA strokes.

**Methods:**

The study was conducted from February 2019 - May 2019 at COMSATS University, Islamabad. A total sample size of N=55 middle cerebral artery ischemic infarct patients was selected with N=29 left middle cerebral artery ischemic infarct patients and N=26 right middle cerebral artery ischemic infarct patients. The sample was assessed on The Neurocognitive Assessment Battery for stroke patients (N-CABS) & The Pittsburgh Sleep Quality Index-Urdu (PSQI-U).

**Results:**

The mean age of the sample was 50.96 years. There was a significant difference among scores of cognitive dysfunction between Left MCA (M=47.28, SD=12.87) and Right MCA stroke patients (M=29.7, S=21.41), t (53) =-6.80, p<0.001. Similarly, there was significant difference among scores of sleep disturbance between Left MCA (M=6.90, SD=2.93) and Right MCA (M=10.35, SD=3.97), t (53) =-3.68, p<0.001. Gender comparisons reveal that there is no significant difference between males and females for both, cognitive dysfunction and sleep quality impairment.

**Conclusions:**

Cognitive dysfunction and sleep quality impairment due to MCA strokes is significant between left and right hemispheres respectively, regardless of gender, assessed with N-CABS and PSQI-U. Further studies are required to analyse other demographic correlates related to MCA strokes.

## INTRODUCTION

A Cerebrovascular accident (CVA) or stroke is clinically defined as a syndrome of rapidly developing symptoms or signs of focal loss of cerebral function with no apparent cause other than that of vascular origin, but the loss of function can at times be global.^1^ The syndrome varies in severity from recovery in a day, through incomplete recovery, to severe disability, to death. More precisely, an ischemic stroke is one which occurs as a result of a blockage or obstruction in an artery carrying blood to the brain. The Middle Cerebral Artery (MCA) is the largest cerebral artery in the anterior region of the brain and also the most common site for CVAs. Neuropsychological outcomes post-stroke are significantly associated with cognitive impairment as well as sleep quality impairment.^1^

Cognitive dysfunction is operationally defined as a decline in cognitive abilities where cognition may be considered as comprising of five primary domains: attention, memory, executive functions, language and visuo-spatial skills. Hemiparesis, sensory deficits, and ataxia can occur with either a right or left hemisphere lesion and typically affect the contralateral side. Speech impairments and aphasias (impairment of language) are more typical with a left hemisphere lesion, while perceptual deficits are more commonly associated with a right hemisphere lesion.^2^ Furthermore, the prevalence of ischemic strokes is almost four times that of hemorrhagic strokes with cognitive dysfunction developing in 25-30% of patients. Cognitive dysfunction in the left hemisphere is more common in strokes while potential recovery prognosis is better for right hemisphere lesions. In studies conducted on the incidence between MCA stroke lateralization specifically, it has also been repeatedly observed that left MCA strokes are more prevalent among the patient population than right MCA strokes.^2^ These differences dictate that there is a need to compare and contrast the hemisphere on neuropsychological variables in order to ascertain their effects. Most studies use Mini Mental state examination but it is insensitive to mild cognitive impairment and executive functions,^3^ hence, a cognitive battery is a more reliable option.

Apart from Post Stroke Cognitive Impairment, sleep quality impairment as a neuropsychological variable has a significant incidence in the acute phase of stroke and it is slightly more common in the right hemisphere than in the left hemisphere. Sleep quality impairment includes sleep breathing problems, sleep disturbances and a higher latency and duration of sleep in acute phase of stroke but a longer sleep duration of patients in the late stage of stroke recovery.^2^ All these, as well as, habitual sleep efficiency, use of sleeping medication, and daytime dysfunction are measured in the Pittsburgh sleep quality index-Urdu (PSQI-U).^4^ These impairments may lead to sleep disorders such as sleep apnea, hypersomnia, insomnia etc. which are not just a consequence of stroke but can also serve as a risk factor for stroke. Literature shows that prevalence of sleep quality impairment as a neuropsychological variable is high in the early phase of stroke and that, to some degree, it is more common in the right hemisphere as compared to the left hemisphere.^2^

Gender differences have also been observed where females generally are more functionally impaired with higher values of sleep quality impairment and cognitive impairment than males.^1^ which indicates that MCA stroke patient will demonstrate a similar pattern however it is yet to be studied.

According to the World Health Organization, Two-thirds of the global deaths associated with strokes occur in developing countries such as Pakistan.^5^ Although no large-scale studies have been conducted to estimate true incidence rates in the country, the Aga Khan University Stroke Data Bank provides that among the risk factors Hypertension and Diabetes Mellitus are most commonly correlated with ischemic stroke cases in Pakistan.^6^ Another study has shown that long-term drug abuse can serve as a risk factor for strokes as well. The changes in the blood flow of the brain caused by the use of synthetic cannabinoids play a role in the development of neurological symptoms that increase the incidence of hemorrhagic or ischemic strokes.^7^ Whereas, sleep needs a more eclectic discussion involving all the dimensions affected by it, that is, psychological, neurological, social and physiological dimensions. All these aspects are affected to some extent when a person is suffering from some sort of illness. It is easier for health professionals to recognize medical causes linked to sleep disturbances in patients, however, it is difficult for them to screen for psychological problems related with sleep issues.^8^

Most of the studies conducted previously used Mental State Examination to study cognitive impairment^6^ whereas the current study has used a cognitive battery, Neurocognitive Assessment Battery for stroke patients (N-CABS) for better assessment of cognitive impairment along with PSQI-U in MCA stroke patients between the left and right hemisphere. Moreover, it will also study gender differences with respect to MCA strokes, which are yet to be studied. The results obtained by this research will be applicable not only in hospital settings for doctors, nurses and mental health professionals but also for family caregivers.

## METHODS

The current study follows a comparative study design as it studies severity of cognitive and sleep quality impairment in left and right MCA stroke patients & between genders. Informed consent, as approved by the Departmental Ethical committee of COMSATS University, Islamabad (Ref. No. CUI-ISB/HUM/ERC-CPA/2019-5 dated July 15, 2019), following American Psychological Association guidelines, was taken from each participant before administering the scales. Purposive sampling technique was employed. The demographics included name, age, gender, education, occupation, past and current medical conditions (including hypertension, diabetes and cardiovascular diseases). The inclusion criteria for participants was age above 18, of either gender, with first time ischemic infarcts in the left or right MCA occurring within the past 12 months and an education level of at least grade 5 (≥ 5th grade). Whereas, patients with history of recurring cerebrovascular accidents (CVAs), diagnosed clinical depression, aphasia, altered state of consciousness were excluded. The data was collected across various in-patient facilities in Hospitals, namely PIMS, CMH, Holy Family and Fauji Foundation Hospital, with permission from February 2019 to May 2019. Krejcie & Morgan^9^ concluded the applicable sample sizes for respective populations by using the formula, s=X^2^NP(1- P)÷d^2^(N1)+X^2^P(1- P) where *s* = required sample size, *X*2 = the table value of chi-square for 1 degree of freedom at the desired confidence level (3.841), *N* = the population size, *P* = the population proportion (assumed to be 0.50 since this would provide the maximum sample size) and *d* = the degree of accuracy expressed as a proportion (0.05). Based on this formula, a sample size of N=56 is required for a population total of 65. The total in-patients with MCA strokes were 65-68 in the above-mentioned hospitals, hence, a total sample of N=55 of middle cerebral artery ischemic infarct patients was selected with N=29 left MCA ischemic infarct patients and N=26 right MCA ischemic infarct patients. The diagnosis of MCA infarct was confirmed by a computed tomography (CT) scan or a magnetic resonance imaging (MRI) scan. Whereas, severity of the stroke was determined through clinical assessment reported by neurologists.

The Neurocognitive Assessment Battery for stroke patients (N-CABS) was developed and validated locally to measure Post Stroke Cognitive Impairment (PSCI).^10^ It was utilized in this study to assess the degree of impairment among the sample. The battery consists of nine sub-tests that include orientation, attention span, paired associate learning test, naming, similarities, arithmetic, Bell’s test, block design and non-verbal learning test. It therefore provides a more comprehensive framework than the MMSE to assess various cognitive faculties in different areas of the brain in association with strokes.^11^ In the current study, the scale’s suitability stems from defined cut off scores indicating the global degree of cognitive impairment resulting from ischemic strokes in the left and right MCA. N-CABS outlines PSCI severity as Suspect (74-82), Mild (64-73), Moderate (55-64), and Severe (<55) with a normal scores above 83.

The Urdu translation of The Pittsburgh Sleep Quality Index (PSQI-U) was employed in this study to measure Subjective Sleep Quality. It is a self-rated questionnaire which assesses sleep quality. It consists of 19 items. The PSQI-U measures several different aspects of sleep, offering seven components scores and one composite score. The component scores consist of subjective sleep quality, sleep latency (i.e., how long it takes to fall asleep), sleep duration, habitual sleep efficiency (i.e., the percentage of time in bed that one is asleep), sleep disturbances, use of sleeping medication, and daytime dysfunction. The Cronbach’s alpha is 0.56.^12^

### Statistical Analysis

SPSS Version 21.0 was used for data analysis. Severity level were reported with percentages. Mean comparison of hemispheric and gender groups was done through independent t-test. P-value less than 0.01 was considered statistically significant.

## RESULTS

In Table-I, a significant majority of the sample falls under the severe category indicating that MCA ischemic infarctions are associated with cognitive impairment. Similarly, in Table-II, frequency analysis shows that 49 patients showed some degree of sleep quality impairment.

**Table-I:** Severity of Cognitive Impairment across MCA patients (N=55).

Severity	Frequency (f)	Percentage (%)
Severe	45	81.8
Moderate	2	3.6
Mild	5	9.1
Suspect	2	3.6
Normal	1	1.8

**Table-II:** Sleep Quality Impairment across MCA patients (N=55).

Sleep Quality Impairment	Frequency (f)	Percentage (%)
Normal	6	10.9
Impaired	49	89.1

A two-tailed t-test was conducted between MCA stroke patients based on hemispheric lateralization of lesion (Table-III). Left MCA stroke patients displayed greater cognitive impairment on the N-CABS (p=0.00) than Right MCA stroke patients. Whereas Right MCA stroke patients received a higher mean rating on sleep quality impairment than left MCA stroke patients (p=0.00). Cohen’s d calculated for both variables on hemispheric comparison showed a very large effect size.

**Table-III:** Hemispheric comparison of cognitive impairment and sleep quality impairment (N=55).

Variable	Left MCA	Right MCA	t (53)	p	95% *Cl*		Cohen’s d
	
	M (SD)	M (SD)			LL	UL	
Cognitive Impairment	47.28(12.87)	49.31 (21.41)	6.80	0.00	-41.47	-22.59	1.81
Sleep Quality Impairment	6.90(2.93)	10.35(3.97)	3.68	0.00	-5.32	-1.57	0.98

***Note:*** **MCA:** Middle Cerebral Artery. **Cl:** Confidence Interval. **M:** Mean. **SD:** Standard Deviation. **t:** t-statistic. **p:** significance. **LL:** Lower Limit. **UL:** Upper Limit.

A two tailed t-test was similarly conducted to determine the difference in means between male and female MCA stroke patient groups (Table-IV). Results indicated that the differences between male and female MCA stroke patients in terms of cognitive and sleep quality impairment post stroke is not significant. This was contrary to what the study postulated i.e. females would show greater impairment on both variables.

**Table-IV:** Gender differences in cognitive impairment and sleep quality impairment (N=55).

Variable	Male	Female	t (53)	p	95% CI	
	
	M (SD)	M (SD)			LL	UL
Cognitive Impairment	35.64(20.39)	29.7 (26.55)	1.031	0.30	-19.34	6.21
Sleep Quality Impairment	9.07(4.05)	7.96(3.61)	1.06	0.29	-3.18	0.97

***Note:*** **MCA:** Middle Cerebral Artery. **Cl:** Confidence Interval. **M:** Mean. **SD:** Standard Deviation. **t:** t-statistic. **p:** significance. **LL:** Lower Limit. **UL:** Upper Limit.

## DISCUSSION

Strokes are one of the major causes of functional disability in the modern world with Ischemic Middle Cerebral Artery (MCA) strokes the most prevalent manifestation in the cerebrovascular system.^1^,^11^,^13^,^14^ The functional impairments most widely associated with these strokes are cognitive impairment^1^ and sleep quality impairment,^15^,^16^ with the former more common in strokes of the left hemisphere^17^ and the latter in strokes of the right hemisphere.^7^ Based on this, the current study postulated strokes in the left MCA would similarly result in greater cognitive impairment while strokes in the right MCA would result in greater sleep quality impairment. Gender was additionally analyzed to provide evidence for the trend of greater female impairment post stroke,^5^,^18^,^19^ and the degree of impairment was also globally assessed.

Among the sample of ischemic MCA stroke patients assessed for degree of impairment, 82% displayed severe degree of cognitive impairment whereas 89% had some level of sleep quality impairment as measured by the N-CABS and the PSQI-U respectively. These levels suggest that cognitive and sleep quality impairment across patients are present in the majority of the cases with high functional consequences. However, the sample primarily included patients from in-patient facilities that receive clinically critical individuals and as such may bias the results.^20^

In a hemispheric comparison between the left and right MCA stroke patients, it was found that left MCA stroke patients were significantly more impaired cognitively than right MCA stroke patients and right MCA stroke patients displayed a greater degree of sleep quality impairment when compared to left MCA stroke patients. These findings are consistent with stroke literature and the large effect sizes further suggest the effect is clearly distinguishable in the population studied. Aphasic dominance in the left MCA group along with the ascribed higher executive role of the left hemisphere may help in elucidating the results on cognitive impairment.^20^-^23^

Differences among genders in a hemispheric MCA stroke comparison yielded non-significant results across both cognitive and sleep quality impairment conditions, suggesting that gender of the patients does not have any substantial effect on post stroke functional impairment. These findings contradict previous literature available on the topic, and while the neuroprotective role of estrogen hormones^24^,^25^ provides support for the results due to the majority of the female cases being premenopausal, it requires further research in order to identify any possible cultural or neurological factors that may have a role in the process.

The current study however employed a relatively simple research design and analytic scheme as a result of access to a limited sample group, which can be addressed in future research. The non-significant result on gender differences across MCA stroke patients also provides an avenue for further research into its potential causes. Furthermore, sleep quality impairment was measured via the PSQI-U which is not designed specifically for the assessment of post stroke sleep quality impairment and as such a more relevant tool may be employed in future studies.

These findings imply significant utility for stroke treatment facilities, where left MCA stroke patients may be more prone to greater degree of cognitive impairment and right MCA stroke patients more prone to greater degree of sleep quality impairment.^18^,^26^ Therefore their treatment and rehabilitation will benefit from the present results.^27^ Furthermore, it details the prevailing trend among MCA stroke patients in in-patient facilities in terms of severity, gender and hemispheric comparisons. All factors that could be valuable in planning a primary and secondary care regimen, management plan and psychological rehabilitation that is adapted specifically to the condition.

### Author`s Contribution:

**DW and HR** conceived, designed and did data collection.

**AI** conceived, did statistical analysis for manuscript and is accountable for the accuracy or integrity of the work.

**ZA** did manuscript writing and editing.
